# *Hungatella hathewayi*: A Tumor-Derived Bacterium Enriched in Colorectal Cancer Tissues and a Potential Diagnostic Biomarker

**DOI:** 10.3390/microorganisms14030707

**Published:** 2026-03-21

**Authors:** Wenzhe Zhang, Jin Liu, Shanshan Sha, Qiulong Yan, Yufang Ma

**Affiliations:** 1 Department of Biochemistry and Molecular Biology, Dalian Medical University, Dalian 116044, China; zhangwz01@dmu.edu.cn (W.Z.); shanshan_sha@dmu.edu.cn (S.S.); 2 Division of Gastrointestinal & Bariatric-Metabolic Surgery, Department of General Surgery, The Second Affiliated Hospital of Dalian Medical University, Dalian 116044, China; liujin285@126.com; 3 Department of Microbiology, Dalian Medical University, Dalian 116044, China

**Keywords:** *Hungatella hathewayi*, intratumoral localization, colorectal cancer, succinate, biomarker

## Abstract

Colorectal cancer (CRC) is the third most common cancer globally and the second leading cause of cancer-related deaths. While intestinal microbiota dysbiosis is linked to CRC, the direct role of intratumoral bacteria in metastasis remains poorly understood. In this study, we isolated pathogenic bacteria from CRC tumor tissues, identified as *Hungatella hathewayi* (*H. hathewayi*), through the 16S rRNA gene and whole-genome sequencing. We developed specific primers (P48/P52) and polyclonal antibodies for detecting *H. hathewayi* in samples. Using quantitative real-time PCR (qPCR), we found significant enrichment of *H. hathewayi* in fecal samples from CRC patients compared to healthy controls, with mean fold changes of 137-fold and 142-fold for primers P48 and P52, respectively. Analysis of tissue samples revealed that *H. hathewayi* abundance was higher in CRC tumor tissues compared to normal tissues, with mean fold changes of 2.90 for P48 and 3.97 for P52. Fluorescence in situ hybridization (FISH), immunofluorescence (IF), and immunohistochemistry (IHC) confirmed its spatial distribution within tumor tissues. In vitro assays using CRC cell lines demonstrated that *H. hathewayi*-derived succinate upregulates *HIF-1α* and *SUCNR1* expression and promotes cell metastasis by inducing epithelial–mesenchymal transition (EMT). Collectively, these findings identify *H. hathewayi* as a novel pro-metastatic bacterium and a potential non-invasive biomarker for CRC diagnosis, providing direct evidence for the role of intratumoral bacteria in CRC progression.

## 1. Introduction

Colorectal cancer (CRC) is a common malignant tumor in the gastrointestinal tract. It accounts for 10% of all malignant tumors and has the third-highest global incidence rate; additionally, it has the second-highest global cancer mortality rate [1]. The rate of decline in CRC incidence decreased from an annual reduction of 3–4% in the 2000s to approximately 1% per year between 2011 and 2019 [2].

Recent scientific estimates suggest that the human gut harbors approximately 40 trillion microbes, a number roughly comparable to the total count of human cells, resulting in a near one-to-one ratio [3]. Intestinal microbiota play a crucial role in digestion, vitamin synthesis, intestinal immunity, and the maintenance of normal intestinal homeostasis [4,5,6]. In recent years, there has been significant interest in the role of microorganisms in carcinogenesis. Various studies have established a connection between disruptions in intestinal microbiota and the onset of CRC. Alterations in intestinal microbiota have been shown to impact the advancement of CRC [7,8]. Bacteria constitute the primary element of the intestinal microbiota, with over 50 bacterial phyla and 1000 bacterial species typically present in normal human intestinal symbiotic microbiota [9,10,11,12]. Studies have indicated that certain intestinal bacteria, such as *Fusobacterium nucleatum* (*F. nucleatum*), genotoxin-producing *Escherichia coli* (*E. coli*), *Bacteroides fragilis* (*B. fragilis*), and *Peptostreptococcus anaerobius* (*P. anaerobius*), significantly contribute to the advancement of CRC [13,14,15,16,17,18,19,20]. However, at present, the research on directly isolating pathogenic strains from CRC tumor tissues and analyzing their pathogenic mechanisms is still relatively limited.

Organic acids, including acetic acid, propionic acid, butyric acid, valeric acid, lactic acid, and succinic acid, are significant metabolites produced by intestinal microorganisms from undigested carbohydrates. Among these, succinate is particularly noteworthy, as it plays a crucial role in conditions such as diabetes-related osteoporosis, rheumatoid arthritis, and CRC [21,22,23].

*Hungatella hathewayi* (*H. hathewayi*) is a Gram-positive, strictly anaerobic, rod-shaped, and spore-forming bacterium [24]. Originally classified as *Clostridium hathewayi*, it was later reclassified into the novel genus *Hungatella* based on the phylogenetic analysis of 16S rRNA gene sequences, whole-genome relatedness, and distinct phenotypic characteristics [24]. *H. hathewayi* has been reported to accumulate in CRC samples [25], which can reduce the sensitivity of CRC cells to 5-FU by reducing CDX2 expression [26], and the use of *H. hathewayi* inoculation can promote the proliferation of mouse colonic epithelial cells [27]. A recent study has further strengthened the association between *H. hathewayi* and CRC. In a pooled analysis of nearly 4000 fecal metagenomes from 18 cohorts, *H. hathewayi* was identified as a reproducible microbial biomarker for CRC, with its abundance consistently elevated in patients [28]. However, there are still limitations regarding the direct isolation of *H. hathewayi* from CRC tumors and exploring how it affects the progression of CRC.

In this study, we isolated a strain of *H. hathewayi* from CRC tumors and also determined the abundance of *H. hathewayi* in the feces and tumors of CRC patients. We investigated the effect of *H. hathewayi* on CRC metastasis, confirming whether *H. hathewayi* can trigger epithelial–mesenchymal transition (EMT) in CRC cells by releasing succinate and promote tumor metastasis or not. The findings may reveal the role of bacterial strain *H. hathewayi* on CRC, and *H. hathewayi* may be used as a potential biomarker for CRC clinical diagnosis.

## 2. Materials and Methods

### 2.1. CRC Tissue and Fecal Samples

Tumor tissues, adjacent non-tumor tissues (<2 cm from tumor margin) and normal tissues (>3 cm from tumor margin) from 28 patients with CRC and fecal samples from 20 patients with CRC were used. Fecal samples from 35 healthy individuals were also included as controls. All patients had primary CRC and had not received any relevant treatment prior to surgery.

### 2.2. Bacterial Culture and Strain Isolation

Under anaerobic conditions, tumor tissue samples were placed into 1.5 mL centrifuge tubes, homogenized with a grinding rod, and mixed well with 1 mL of normal saline. The homogenates were serially diluted 10-fold with sterile saline to obtain dilutions of 10^−1^ to 10^−6^. The 50 μL of the each diluent was spread on solid anaerobic medium (GAM, Beijing Luqiao Technology Co., Ltd. (Beijing, China); composition per liter: peptone 15 g, yeast extract 5 g, soy peptone 5 g, beef extract 5 g, glucose 5 g, NaCl 5 g, soluble starch 3 g, L-cysteine 0.5 g, KH_2_PO_4_ 2.5 g, hemin 0.005 g, vitamin K_1_ 0.001 g, and pH 7.3 ± 0.2). All plates were incubated in an anaerobic incubator (LAI-3T, Longyue, Shanghai, China) at 37 °C for 24–72 h under an atmosphere of 90% N_2_, 5% CO_2_, and 5% H_2_. A sterility control plate (incubated without inoculation) was included in each batch to monitor contamination. A single colony was inoculated into liquid anaerobic medium for extended culture under the same anaerobic conditions at 37 °C for 24–48 h.

### 2.3. Bacterial Identification by 16S rRNA Gene

The genomic DNA was prepared using Bacterial Genomic DNA Extraction Kit (TianGen Biotechnology Co., Ltd., Beijing, China). The 16S rRNA gene was amplified by PCR using primers 7F (5′-AGAGTTTGATYMTGGCTCAG-3′) and 1510R (5′-ACGGYTACCTTGTTACGACTT-3′), targeting the full-length 16S rRNA gene. PCR amplification was performed in a thermal cycler (TC-96, Bioer Technology Co., Ltd., Hangzhou, China) using 2 × Accurate Taq Master Mix (Accurate Biology, Changsha, China; cat. no. AG11019). A negative control (sterile distilled water instead of DNA template) was included in each PCR run to monitor contamination. The thermal cycling conditions were as follows: initial denaturation at 94 °C for 5 min, followed by 35 cycles of 94 °C for 25 s, 52 °C for 20 s, and 72 °C for 1 min 45 s, and a final extension at 72 °C for 5 min. The amplified PCR products were verified by 1% agarose gel electrophoresis and then sequenced by General Biol Co., Ltd. (Chuzhou, China). The obtained sequences were compared against the NCBI nucleotide database using BLAST + 2.17.0. The 16S rRNA gene sequence of the isolate showed the highest similarity (≥99%) to that of the *Hungatella hathewayi* DSM 13479 (GenBank accession no. CP102274.1), confirming its species identification. The entire genome was sequenced by Wuhan Puensum Biotech Co., Ltd. (Wuhan, China).

### 2.4. Extraction of Bacterial Genomes from Tissues and Feces

Approximately 25–30 mg of CRC tissues was placed into grinding tubes containing grinding beads (Servicebio, Wuhan, China, G0202-150G,) and 200 μL of 0.9% (*w*/*v*) NaCl and then homogenized using a cryogenic grinder (Wuhan Servicebio Biotechnology Co., Ltd., Wuhan, China; 70 Hz, 30 s work, 15 s internal, total of 4 cycles) at −40 °C. For fecal samples, 180–220 mg was used for DNA extraction. Genomic DNA was extracted from homogenate tissue and fecal samples using TIANamp Stool DNA Kit (TianGen Biotechnology Co., Ltd., Beijing, China) according to the protocol. DNA concentration was measured using a NanoDrop 2000 (Thermo Fisher Scientific, Waltham, MA, USA), and DNA purity was assessed with the A260/A280 ratio, which ranged from 1.8 to 1.9. An extraction blank control (using only lysis buffer) was processed in parallel with each batch of samples to monitor for contamination during the extraction process.

### 2.5. H. hathewayi Abundance Detection

Based on the genome data of *H. hathewayi*, two sets of specific primers P48 and P52 were designed to detect the relative abundance of *H. hathewayi* in fecal samples and tissues of CRC by quantitative real-time PCR (qPCR). To exclude potential interference from host DNA, universal bacterial primers targeting the 16S rRNA gene (16S-1 and 16S-2) were used to quantify total bacterial load, as they specifically amplify bacterial DNA without cross-reactivity with human genomic DNA. The cycle threshold (Ct) value, defined as the number of PCR cycles required for the fluorescent signal to exceed the background threshold, was recorded for each reaction. The mean Ct value of the three replicates was calculated for each sample and used for further analysis. The relative abundance of *H. hathewayi* was determined through normalization of the total bacterial load of the corresponding sample. This was achieved using the 2^−^^ΔCt^ method, where ΔCt was calculated as the difference between the mean Ct value of *H. hathewayi* (using P48/P52 primers) and the mean Ct value of the total bacterial control (16S rRNA gene). Results were expressed as relative units. The sequences of all primers are as follows: P48-1 (forward): 5′-GAGGAGTTGCTATCTTTGT-3′; P48-2 (reverse): 5′-AGGTCATTCTTCATCTTCATA-3′; P52-1 (forward): 5′-CGGAATCAAACAGACAAA-3′; P52-2 (reverse): 5′-GCAGTAGGAATCGCTATT-3′; 16S-1 (forward): 5′-CTCCTACGGGAGGCAGCAG-3′; and 16S-2 (reverse): 5′-TTACCGCGGCTGCTGGCAC-3′.

### 2.6. Preparation of Polyclonal Antibody to H. hathewayi

Polyclonal antibody was generated for *H. hathewayi* through immunizing mice in our lab. Bacteria with 10^8^ colony-forming unit (CFU) were collected, resuspended in 1 mL sterile PBS, and sonicated using JY92-IIN ultrasonic cell disruptor (Scientz Biotechnology Co., Ltd., Ningbo, China; 400 W, 10 s work, 10 s internal, total work time of 2 min) on ice to break the bacteria. The lysis efficiency was confirmed by inoculating the bacterial lysate on GAM agar plates and growing them in anaerobic conditions at 37 °C for 48 h. The disrupted bacteria were then mixed in equal volumes with a fluorine adjuvant (F588, Sigma, St. Louis, MO, USA). Eight 7-week-old female Balb/c mice were selected and each mouse received subcutaneous injection of 10^7^ CFU bacteria. A total of four immunizations were administered, with the initial immunization followed by subsequent doses every seven days. Seven days after the final immunization, blood samples were collected from the mice’s eyes without anesthesia. The mice were sacrificed by cervical dislocation immediately after blood collection. About 0.5–0.8 mL blood samples were obtained from each mouse, which were allowed to stand at room temperature for 2 h before being centrifuged at 2500 rpm for 10 min to separate the serum. The separated serum was aliquoted and stored at −80 °C. The specificity of polyclonal antibody in the serum against *H. hathewayi* was tested by Western blot, using *P. anaerobius* and *E. coli* as controls.

### 2.7. Fluorescence In Situ Hybridization (FISH)

Frozen tissues were incubated overnight in the in situ hybridization fixative (Wuhan Servicebio Biotechnology Co., Ltd., Wuhan, China) to prepare 3 mm thick sections for hybridization. *H. hathewayi*-specific P48-1 sequence, 5′-GAGGAGTTGCTATCTTTGT-3′, was labeled at its 5′ end as Cy5-labeled green probe (General Biol Co., Ltd., Chuzhou, China). The bacterial universal fluorescent probe EUB338, a 5′Cy3-labeled red probe with the sequence 5′-GCTGCCTCCCGTAGGAGT-3′ (General Biol Co., Ltd., Chuzhou, China) was utilized alongside it. Following the completion of hybridization, the hybridization solution was removed, and DAPI staining solution along with an anti-fluorescence quenching mounting medium (Wuhan Servicebio Biotechnology Co., Ltd., China) was applied for mounting. The mean fluorescence intensity of *H. hathewayi*-specific signals was measured using ImageJ software (version 1.8.0). Fold changes relative to normal tissues were determined, and the data were from three independent experiments.

### 2.8. Immunohistochemistry (IHC)

The normal tissues from CRC patients were used as blank controls, and the normal tissues injected with *H. hathewayi* were positive controls. Paraffin-embedded sections were dewaxed in xylene (2 × 7 min), rehydrated through graded ethanol (100–70%, 5 min each), and washed with distilled water. Antigen retrieval was performed by microwave heating in retrieval solution (AbCracker, HISTOVA, Beijing, China; cat. no. ABCFR5L) diluted 1:50 in distilled water (10 min at sub-boiling), followed by natural cooling. After PBS washing, endogenous peroxidase was blocked with 3% H_2_O_2_ for 10 min at room temperature. Sections were again PBS-washed (3 × 5 min) and incubated with blocking solution (HISTOVA, Beijing, China; cat. no. GTBB30) for 30 min at 37 °C. Primary antibody (*H. hathewayi* polyclonal antibody) diluted at 1:300 in antibody diluent (HISTOVA, Beijing, China; cat. no. ADB50) was applied overnight at 4 °C in a humidified chamber. After PBS washing (3 × 5 min), sections were incubated with horseradish peroxidase-conjugated sheep anti-mouse IgG polymer (ZSGB-BIO, Beijing, China; cat. no. PV-6000D) as the secondary antibody for 30 min at 37 °C. Following another PBS wash (3 × 5 min), immunoreactivity was visualized using a DAB substrate kit (ZSGB-BIO, Beijing, China; cat. no. PV-6000D) applied for 5 min at room temperature and terminated with distilled water. Sections were counterstained with hematoxylin for 2 min, differentiated in hydrochloric acid ethanol for 5 s, and rinsed in tap water for 5 min. Sections were dehydrated through graded ethanol (70–100%, 5 min each), cleared in xylene (2 × 7 min), and mounted with mounting medium (CrystalMount, HISTOVA, Beijing, China; cat. no. CMT110). The mean staining intensity of *H. hathewayi*-positive signals was measured using ImageJ software. Fold changes relative to normal tissues were determined, and the data were from three independent experiments.

### 2.9. Immunofluorescence (IF)

The experimental groups were the same as those described for IHC. The initial procedures for overnight blocking are same as those of IHC. The polyclonal antibody to *H. hathewayi* (dilution ratio 1:800) was applied in a wet box at 4 °C overnight. Subsequently, the poly-HRP-conjugated goat anti-mouse secondary antibody (product no. PHGM15, Haosai Tuohua Biotechnology Co., Ltd., Beijing, China) was added dropwise and incubated at 37 °C for 30 min. Following this, a PBS wash was performed, and the chromogenic solution was added dropwise, allowing for a 5 min reaction at room temperature. The reaction was then terminated, followed by rinsing with distilled water, and sealing with DAPI. The mean fluorescence intensity of *H. hathewayi*-specific signals was measured using ImageJ software. Fold changes relative to normal tissues were determined, and the data were from three independent experiments.

### 2.10. Succinate Detection

The *H. hathewayi* was cultured in GAM liquid medium at 37 °C under anaerobic conditions for 48 h. The culture medium supernatant of *H. hathewayi* (HHM) in the stationary phase was collected and sent to Suzhou PANOMIX Biomedical Tech Co., Ltd. (Suzhou, China) for targeted detection of succinic acid by LC-MS/MS. GAM is the control group.

### 2.11. Thin-Layer Chromatography (TLC)

Following the collection of the HHM, it was freeze-dried using a freeze dryer (Genscience Eco mini -85, Nanjing Jinshi Instrument Equipment Co., Ltd., Nanjing, China). The resulting freeze-dried powder was then dissolved in distilled water and applied to the silica gel 60 plate (1.06553.0001, Merck, Rahway, NJ, USA). Succinate (14160, Sigma-Aldrich, St. Louis, MO, USA) served as a standard. After natural air drying, the TLC plate was immersed in a developing solution composed of ethanol, ammonium hydroxide, and water (20:5:3 *v*/*v*). After the solution was spread out on the plate, the plate was air-dried and subsequently sprayed with a 4% (*w*/*v*) bromocresol green solution in ethanol. The plate was then heated at 160 °C for 5 min to visualize the organic acid spots.

### 2.12. Cell Culture

CRC cell lines SW620 and HCT15 were selected for the experiment. SW620 was cultured in Dulbecco’s Modified Eagle Medium (DMEM) (11965092, Gibco, Thermo Fisher Scientific, Waltham, MA, USA) containing 10% fetal bovine serum (FBS) and 1% penicillin and streptomycin, and HCT15 was cultured in RPMI 1640 (11875119, Gibco, Thermo Fisher Scientific, USA) containing 10% FBS and 1% penicillin and streptomycin.

### 2.13. CCK-8

For the co-culture of bacteria and cells, SW620 cells were subjected to treatment with *H. hathewayi* under anaerobic conditions at varying multiplicities of infection (MOI). Cells were infected at MOIs of 0.1, 0.5, 1, 3, 5, 10, 20, 50, and 100 for 4 h. Following infection, the cells were washed three times with PBS and subsequently transferred to cell culture medium for an additional 24 h. The absorbance was measured using the Cell Counting Kit-8 (SC119-01, CCK-8, Seven Innovation Biotechnology Co., Ltd., Beijing, China). For the co-culture of bacterial lysate and cells, HCT15 and HepG2 cells were seeded in 96-well plates at a density of 10^4^ cells per well and incubated with cell culture medium containing 5% bacterial lysate for 24 h. For the co-culture of culture medium supernatant with cells, HCT15 cells were treated with cell culture media containing HHM, GAM (0.5%, 5%, 10%, and 20%) or succinate (SA, 0.5 mM, 1 mM, 2.5 mM, and 5 mM).

### 2.14. Transwell Assay

For cell migration experiments, based on a different treatment, 5 × 10^4^ cells were seeded into the upper chamber of cell culture inserts (24-well, 8.0 μm pore size, JET BIOFIL, cat. no. TCS020024, Guangzhou, China) and cultured in the medium without FBS containing 5% volume of bacterial cell lysis, 0.5% and 5% HHM or GAM, and 1 mM succinate (positive control). Following a 48 h incubation at 37 °C in a cell culture incubator, the migrated cells were fixed using 4% paraformaldehyde and then stained with a 0.1% crystal violet solution. Subsequently, the dye was removed with water, and images were captured and documented under a microscope. For cell invasion experiments, cells were seeded into the upper chamber inserts, which were pre-coated with Matrigel (ECM Gel from Sigma-Aldrich, Cat. No. E1273). All other experimental procedures were identical to those used in the migration assay.

### 2.15. RNA Extraction and cDNA Synthesis

SW620 cells were co-cultured with *H. hathewayi* at an MOI of 20:1 for 24 h. HCT15 cells were cultured in cell medium with 5% HHM, GAM, and 1 mM succinate (positive control) for 24 h. Total RNA was extracted from SW620 and HCT15 cell lines, respectively, according to the protocol accompanying the Vazyme FreeZol Reagent (Nanjing, China, cat. no. R711-02). Cell culture medium was removed, and cells were washed once with PBS. For each well of a 6-well plate, 500 μL of FreeZol Reagent was added, and cells were dislodged by repeated pipetting. The lysate was transferred to a 1.5 mL microcentrifuge tube, vortexed thoroughly, and incubated at room temperature for 5 min. A total of 150 μL of Dilution Buffer was added, vortexed, and incubated for another 5 min. The mixture was centrifuged at 12,000× *g* for 15 min at room temperature, and the supernatant was transferred to a fresh tube. An equal volume of isopropanol was added, mixed by inversion, and incubated for 10 min. After centrifugation at 12,000× *g* for 10 min, the supernatant was discarded, and the RNA pellet was retained. The pellet was washed twice with 1 mL of 75% ethanol, each followed by centrifugation at 8000× *g* for 3 min. The supernatant was removed, and the pellet was air-dried. Total RNA was dissolved in 20 μL of RNase-free H_2_O. RNA integrity was assessed by 1% agarose gel electrophoresis, and concentration and purity were determined using a Nanodrop 2000 (Thermo Fisher Scientific, USA) spectrophotometer (A260/280 ratios 1.8–2.1), confirming no degradation or contamination. cDNA was synthesized with the Accurate Biology Evo M-MLV RT Kit (AG11705, Changsha, China) according to the supplied protocol. gDNA Clean Reagent (1 μL), 5 × gDNA Clean Buffer (2 μL), and total RNA (1 μg) were added to a reaction tube, and the volume was adjusted to 10 μL with RNase-free H_2_O. The mixture was incubated at 42 °C for 2 min to remove genomic DNA. Evo M-MLV RTase Enzyme Mix (1 μL), Oligo dT (18T) Primer (50 μM, 1 μL), Random 6 mers Primer (400 μM, 1 μL), 5 × RTase Reaction Buffer Mix I (4 μL), and RNase-free H_2_O (3 μL) were added to the above reaction mixture, resulting in a total volume of 20 μL. The mixture was incubated in the PCR thermal cycler (TC-96, Bioer Technology Co., Ltd., Hangzhou, China) at 37 °C for 15 min, followed by 85 °C for 5 s to complete the cDNA synthesis.

### 2.16. Quantitative Real-Time PCR (qPCR)

For *H. hathewayi* abundance detection, qPCR was performed using the ChamQ Blue Universal SYBR qPCR Master Mix (Vazyme, Nanjing, China, cat. no. Q312-02) on a BIOER FQD-96A system (Hangzhou, China). Each 20 μL reaction contained 10 μL of 2 × Master Mix, 200 nM each of forward and reverse primers, and 40–80 ng of extracted fecal or tissue DNA. The thermal cycling conditions were as follows: 95 °C for 30 s, followed by 45 cycles of 95 °C for 10 s and 60 °C for 30 s. The primers for detecting *H. hathewayi* abundance are described in the corresponding section. For the detection of *NLRP3*, *IL-1β*, *ASC*, *Caspase-1*, *IL-18*, *SUCNR1*, *HIF-1α*, *CDH1*, *Vimentin*, *Snail1*, and *β-actin* mRNA expression, qPCR was performed using the same reagents and thermal cycling conditions as described above. Untreated cells served as the blank control. Target gene expression relative to *β-actin* was calculated using the 2^−^^ΔΔCt^ method. ΔΔCt was determined by subtracting the mean ΔCt of the blank control group from the ΔCt of the treated group, where ΔCt = Ct (target gene) − Ct (*β-actin*). The sequences of all primers are provided in Table 1.

### 2.17. Western Blot

For Western blot, SW620 cells were co-cultured with *H. hathewayi* or *F. nucleatum* (positive control) at an MOI of 20:1 for 24 h. HCT15 cells were cultured in cell medium with 5% HHM, GAM and 1 mM succinate (positive control) for 24 h. Total protein was extracted from SW620 and HCT15 cells using the Whole Protein Extraction Kit (KeyGEN BioTECH, Nanjing, China, cat. no. KGB5303), according to the manufacturer’s protocol. Protein concentration was determined using the BCA Protein Quantification Kit (Vazyme, Nanjing, China, cat. no. E112-01), following the manufacturer’s instructions. Equal amounts of protein were loaded per lane: 120 μg for NLRP3 and Caspase-1 detection, and 30 μg for other target proteins. Proteins were separated on 10% to 15% SDS-PAGE and then transferred to nitrocellulose membrane using a HT-ZY03 Mini Transfer Electrophoresis Unit (Hongji Biotechnology Co., Ltd., Nanjing, China) at a constant current of 260 mA for 1 h (wet transfer). The membranes were blocked with 10% non-fat milk in TBST for 3 h at room temperature. The membranes were incubated overnight at 4 °C with primary antibodies targeting NLRP3 (Cat No. 30109-1-AP, ProteinTech Group, Inc., Rosemont, IL, USA), IL-1β (516288, ZEN-BIOSCIENCE Co., Ltd., Chengdu, China), ASC (Cat No. 10500-1-AP, ProteinTech Group, Inc.), Caspase-1 (Cat No. 22915-1-AP, ProteinTech Group, Inc.), IL-18 (Cat No. 10663-1-AP, ProteinTech Group, Inc.), HIF-1α (Cat. PB9253, Boster Biological Technology Co., Ltd., Wuhan, China), CDH1 (Cat. PTM-6222, PTM BioLab Inc., Hangzhou, China), Vimentin (Cat. PTM-5376, PTM BioLab Inc.), Snail1 (Cat. 3879, Cell Signaling Technology, Inc., Danvers, MA, USA), and β-actin (Cat. PTM-5455, PTM BioLab Inc.), followed by a 1 h incubation with secondary antibodies (Cat No. SA00001-2, ProteinTech Group, Inc.) at room temperature. After washing, the membranes were visualized using Ultra High Sensitivity ECL Kit (GLPBIO, Montclair, CA, USA; cat. no. GK10008).

### 2.18. Statistical Analysis

Statistical analyses were performed using GraphPad Prism version 10.1.2. Data were obtained from three independent experiments and presented as mean ± SD. The normality of data distribution was assessed using the Shapiro–Wilk test. For datasets that conformed to a normal distribution, comparisons between two groups were conducted using unpaired Student’s *t*-test. For datasets that did not conform to a normal distribution, the non-parametric Mann–Whitney test was used instead. For bacterial abundance among different tissues derived from the same patient (matched design), Repeated Measures One-Way Analysis was performed, followed by Dunnett’s multiple comparison test, with normal tissues serving as the control. For other multiple group comparisons (unmatched design), standard One Way ANOVA was performed, followed by Dunnett’s multiple comparison test against the blank control group. The diagnostic performance of *H. hathewayi* in fecal samples for predicting CRC was evaluated by receiver operating characteristic (ROC) curve analysis. The area under the curve (AUC) was calculated with 95% confidence interval (CI). The optimal cutoff value was determined using Youden’s index (maximum sensitivity + specificity − 1), and the corresponding sensitivity and specificity were calculated. *p*-value < 0.05 was considered statistically significant.

## 3. Results

### 3.1. H. hathewayi Is Highly Abundant in CRC Patients and Within CRC Tumors

**Figure 1:**
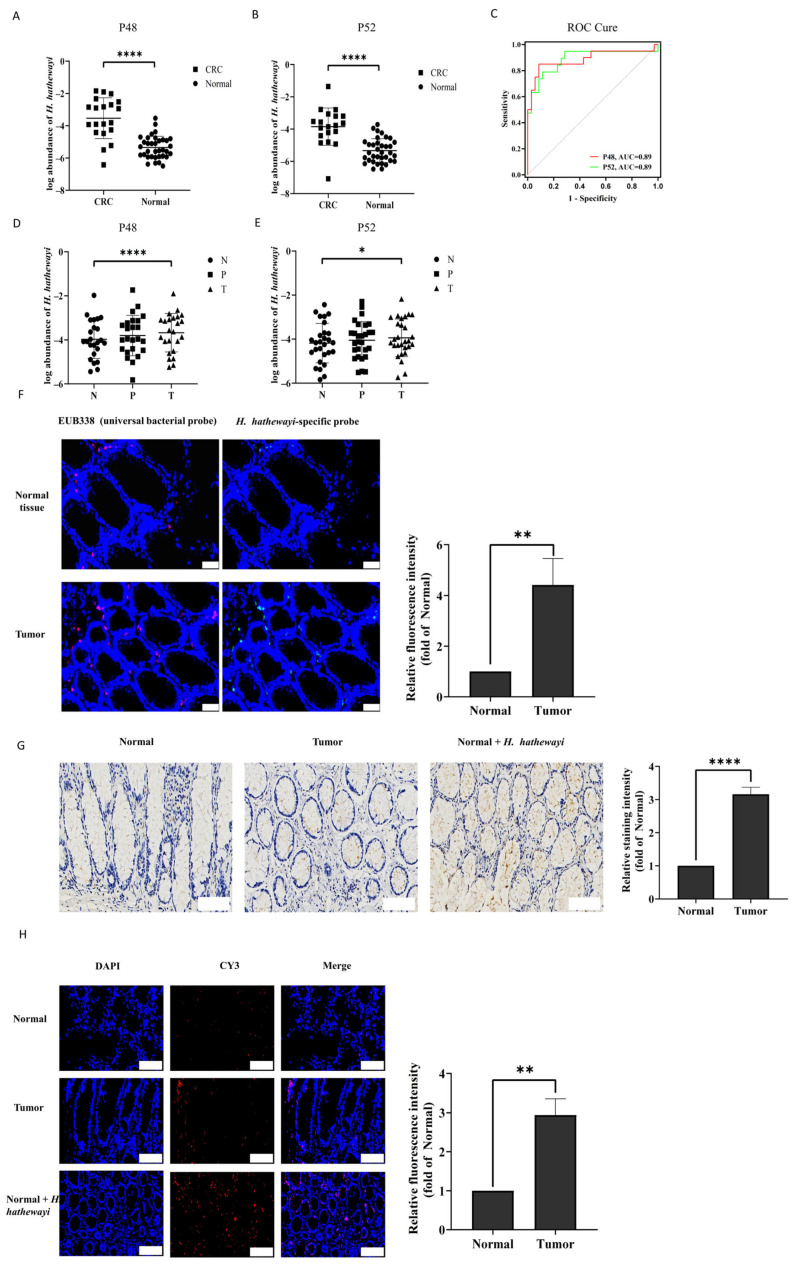
*H. hathewayi* is highly abundant in CRC patients and within CRC tumors. (**A**,**B**) Quantification of the abundance of *H. hathewayi* in healthy subjects (Normal) and CRC patient fecal samples using specific primers P48 (**A**) and P52 (**B**) by qPCR. Primer set P48 was used to analyze 35 healthy subject fecal samples and 20 CRC patient fecal samples, whereas primer set P52 was used to analyze 35 healthy subject fecal samples and 19 CRC patient fecal samples. Data are shown on a log_10_ scale. (**C**) Receiver operating characteristic ROC curve analysis of fecal *H. hathewayi* abundance for discriminating CRC patients from healthy individuals. The diagnostic performance of *H. hathewayi* detected by specific primers, P48 (red) and P52 (green), was evaluated. The area under the curve (AUC) was calculated with 95% confidence interval (CI). For primer P48, the AUC was 0.887 (95% CI: 0.777–0.997). For primer P52, the AUC was 0.887 (95% CI: 0.777–0.997). (**D**,**E**) Quantification of the relative abundance of *H. hathewayi* in tissue samples using specific primers P48 (**D**) and P52 (**E**). Primer set P48 was used to analyze 24 tissue samples from CRC patients, including normal tissues (N), adjacent non-tumor tissues (P), and tumor tissues (T), while primer set P52 was applied to 28 such samples. Data are shown on a log_10_ scale. (**F**) FISH detection was performed using a Cy3-conjugated universal bacterial probe targeting 16S rRNA (EUB338, red) and Cy5-conjugated *H. hathewayi* probe (green). Scale bars, 50 µm. (**G**) Localization of *H. hathewayi* in tissues by IHC. The normal tissues from CRC patients (Normal) were used as blank controls, and the normal tissues injected with *H. hathewayi* (Normal + *H. hathewayi*) were positive controls. Scale bars, 100 µm. (**H**) Localization of *H. hathewayi* (red) in CRC tissues by IF. The normal tissues from CRC patients (Normal) were used as blank controls, and the normal tissues injected with *H. hathewayi* (Normal + *H. hathewayi*) were positive controls. Scale bars, 100 µm. Results were reproducible across three independent experimental replicates. Data were from independent experiments and shown as mean ± SD. Compared using Student’s *t*-test between two groups, and differences among the groups were calculated using One Way ANOVA, followed by Dunnett’s multiple comparison test. * *p* < 0.05, ** *p* < 0.01 and **** *p* < 0.0001.

To determine whether there was a high abundance of *H. hathewayi* in CRC patients, we collected fecal samples from healthy subjects (Normal) and CRC patients. The relative abundance of *H. hathewayi* in fecal samples was detected by qPCR using the specific primers P48 and P52 of *H. hathewayi*. The qPCR results revealed that the mean abundance of *H. hathewayi* was significantly higher in fecal samples from CRC patients compared to healthy controls (Figure 1A,B). The relative abundance in CRC patients compared with controls was 137-fold and 142-fold for the primers P48 and P52. To evaluate the potential of fecal *H. hathewayi* abundance as a non-invasive diagnostic biomarker for CRC, we performed receiver operating characteristic (ROC) curve analysis. As shown in Figure 1C, the area under the curve (AUC) for *H. hathewayi* detected by primer P48 was 0.887 (95% confidence interval: 0.777 to 0.997), with a sensitivity of 85% and a specificity of 91.43% at the optimal cutoff value of 3.19 × 10^−5^. Similarly, for primer P52, the AUC was 0.887 (95% confidence interval: 0.777 to 0.997), yielding a sensitivity of 78.95% and a specificity of 88.57% at the cutoff value of 3.55 × 10^−5^. These results suggest that fecal *H. hathewayi* abundance has promising diagnostic accuracy for distinguishing CRC patients from healthy individuals. To further validate the diagnostic performance of the selected cutoff values, we evaluated them in an independent cohort using both P48 and P52 primers. For primer P48, the cutoff value of 3.55 × 10^−5^ yielded a sensitivity of 85.0% (17/20) and a specificity of 91.43% (32/35) in the validation cohort, which is identical to the discovery cohort (Supplementary Table S1). For primer P52, the cutoff value of 3.55 × 10^−5^ achieved a sensitivity of 78.95% (15/19) and a specificity of 88.57% (31/35), also matching the discovery cohort performance (Supplementary Table S2). These results demonstrate the robustness and generalizability of fecal *H. hathewayi* as a diagnostic biomarker for CRC.

To further investigate the distribution of *H. hathewayi* in tissues, the abundance of differences in *H. hathewayi* in CRC tumor tissues, adjacent non-tumor tissues, and normal tissues were also detected by qPCR using the same primers. The results indicated that, compared with normal tissues, the relative abundance of *H. hathewayi* was higher in tumor tissues, with a mean fold change of 2.90 for the P48 and 3.97 for the P52. However, there was no significant difference between adjacent tissues and tumor tissues (Figure 1D,F). We further compared the abundance of *H. hathewayi* in tumor tissues from patients with early-stage (stage I–II) and late-stage (stage III–IV) CRC. As shown in Supplementary Figure S1, qPCR analysis using both P48 and P52 primers revealed that *H. hathewayi* abundance showed no significant difference between early-stage and late-stage CRC tumor tissues. This finding indicates that the enrichment of *H. hathewayi* occurs at an early-stage of tumorigenesis and persists throughout tumor progression, suggesting its potential role as an early event in CRC development rather than a late-stage phenomenon.

To further confirm the tissue localization of *H. hathewayi*, we used *H. hathewayi* oligonucleotide probes to perform fluorescence in situ hybridization (FISH) between the tumor tissues and normal tissues of CRC patients. As shown in Figure 1F, the red fluorescence signals represent total bacteria, while the green signals specifically label *H. hathewayi*. The results showed that *H. hathewayi* was significantly higher in CRC tumor tissues than in normal tissues, with a mean fold change of 4.42. Meanwhile, the polyclonal antibody targeting *H. hathewayi* (its specificity is in Supplementary Figure S2) was employed to determine the localization of *H. hathewayi* in tumors through IF and IHC experiments. The normal tissues (Normal) from CRC patients were used as blank controls, and the normal tissues injected with *H. hathewayi* (Normal + *H. hathewayi*) were positive controls. IHC staining confirmed that *H. hathewayi* was enriched in tumor tissues, and the relative staining intensity revealed that the amount of *H. hathewayi* in tumor tissues was significantly higher, with a mean fold change of 3.16 compared to normal tissues (Figure 1G). Similar to the IHC results, positive controls showed clear labeling of *H. hathewayi* with red fluorescence, and IF staining further confirmed the enrichment of *H. hathewayi* in tumor tissues, with a mean fold change of 2.94 in relative fluorescence intensity compared to normal tissues (Figure 1H). Collectively, these results indicate that *H. hathewayi* is highly abundant in the feces of CRC patients and is also enriched in CRC tumors.

### 3.2. H. hathewayi Induces Inflammasome Activation in Intestinal Epithelial Cells

**Figure 2:**
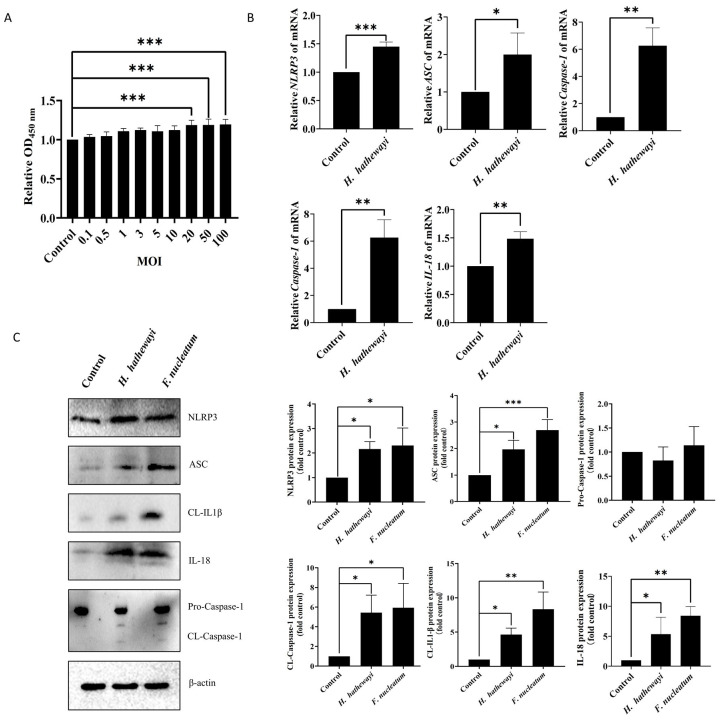
*H. hathewayi* induces inflammasome activation in intestinal epithelial cells. (**A**) SW620 cells were co-cultured with *H. hathewayi* at different multiplicities of infection (MOI) for 4 h, washed with PBS, and then incubated in fresh cell culture medium for 24 h. Cell proliferation was assessed by CCK-8 assay. Untreated SW620 cells served as the control group. (**B**) Relative mRNA expression levels of pro-inflammatory factor genes (*NLRP3*, *ASC*, *IL-1β*, *IL-18*, and *Caspase-1*) in SW620 by qPCR. Cells were infected with *H. hathewayi* at MOI 20:1 for 24 h. Untreated cells served as the blank control. (**C**) SW620 cells were co-cultured with *H. hathewayi* or *F. nucleatum* (positive control) at MOI 20:1 for 24 h. Protein levels of NLRP3, ASC, pro-Caspase-1, cleaved-Caspase-1 (CL-Caspase-1), IL-18, and cleaved-IL-1β (CL-IL-1β) were detected by Western blot, with β-actin as the reference protein. Data were from independent experiments and shown as mean ± SD. Compared using Student’s *t*-test between two groups, and differences among the groups were calculated using One Way ANOVA, followed by Dunnett’s multiple comparison test. * *p* < 0.05, ** *p* < 0.01, and *** *p* < 0.001.

To determine the effect of *H. hathewayi* on intestinal epithelial cells, we co-cultured *H. hathewayi* with SW620 cells under varying multiplicity of infection (MOI) conditions by CCk-8. The findings indicated that *H. hathewayi* significantly enhanced SW620 cell proliferation at MOIs of 20:1, 50:1, and 100:1 (Figure 2A). Additionally, at an MOI of 20:1, *H. hathewayi* enhanced the mRNA expression levels of *NLRP3*, *ASC*, *Caspase-1*, *IL-1β*, and *IL-18* in SW620 cells (Figure 2B). Subsequent Western blot analysis confirmed that *H. hathewayi* increased the protein levels of NLRP3, ASC, cleaved-Caspase-1, IL-1β, and IL-18 (Figure 2C). Our results demonstrate that *H. hathewayi* infection activates Caspase-1 via the NLRP3/ASC inflammasome, leading to the release of IL-1β and IL-18, and thereby triggering a local inflammatory response in intestinal epithelial cells.

### 3.3. H. hathewayi Bacterial Lysate Enhances the Proliferation, Migration, and Invasion of CRC Cells

**Figure 3:**
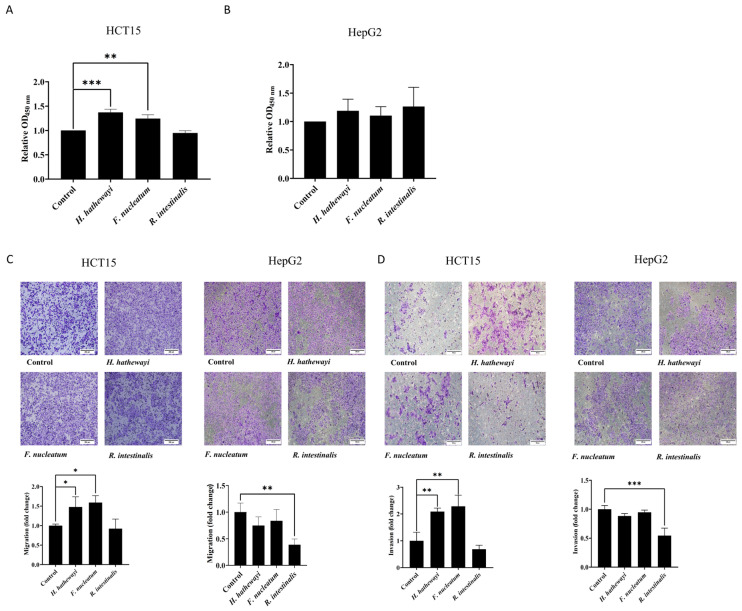
*H. hathewayi* bacterial lysate enhances the proliferation, migration, and invasion of CRC cells. (**A**,**B**) HCT15 (**A**) and HepG2 (**B**) cells were seeded in 96-well plates at a density of 10^4^ cells per well. Cells were treated with 5% lysate from *H. hathewayi*, *F. nucleatum* (used as a positive control), or *R. intestinalis* (negative control) for 24 h. Untreated cells served as the blank control. Cell viability was analyzed by CCK-8 assay. (**C**) HCT15 and HepG2 cells seeded in the upper chamber of the transwell plates were treated with 5% lysates from *H. hathewayi*, *F. nucleatum* (positive control), or *R. intestinalis* (negative control) in serum-free medium for 24 h for migration analysis. Untreated cells served as blank controls. The number of migrated cells is presented as fold change relative to the control group. Scale bars, 200 μm. (**D**) Cell invasion was analyzed using Matrigel-coated inserts. All other experimental conditions were as described in (**C**). Scale bars, 200 μm. Data were from independent experiments and shown as mean ± SD. Differences among the groups were calculated using One Way ANOVA, followed by Dunnett’s multiple comparison test. * *p* < 0.05, ** *p* < 0.01, and *** *p* < 0.001.

As we have determined the colonization and high abundance of *H. hathewayi* within the tumor tissues of CRC patients, we hypothesized that *H. hathewayi* may have a positive effect on the development of CRC tumors. Next, we added *H. hathewayi* bacterial lysate to the human CRC cell line HCT15 and human hepatoma cell line HepG2 to observe the effects of *H. hathewayi* on the proliferation, migration, and invasion of CRC cells. As shown in Figure 3A,B, cell viability measured by CCK-8 assay showed that *H. hathewayi* lysate significantly promoted the proliferation of HCT15 cells compared to untreated cells (Control), with effects similar to the positive control, *F. nucleatum* lysate. In contrast, the negative control, *Roseburia intestinalis* (*R. intestinalis*) lysate, did not affect HCT15 cell proliferation (Figure 3A). No significant effect on HepG2 cell proliferation was observed with any of the bacterial lysates when compared to untreated cells (Figure 3B). Furthermore, migration assays using transwell revealed that the *H. hathewayi* lysate significantly enhanced the migration of HCT15 cells, with the number of migrated cells presented as a fold change relative to the untreated cells (Control). Specifically, *H. hathewayi* treatment resulted in a 1.5-fold increase in HCT15 cell migration compared to control groups, while no effect was seen on HepG2 cells (Figure 3C). Similarly, invasion assays demonstrated that *H. hathewayi* lysate promoted the invasion of HCT15 cells but not HepG2 cells, with data expressed as a fold change relative to the untreated cells (Control). *H. hathewayi* treatment led to a 2-fold increase in HCT15 cell invasion compared to the untreated cells (Figure 3D). Together, these results demonstrate that *H. hathewayi* lysate promotes the proliferation, migration, and invasion of CRC cells.

### 3.4. H. hathewayi-Derived Succinate Could Promote CRC Metastasis

**Figure 4:**
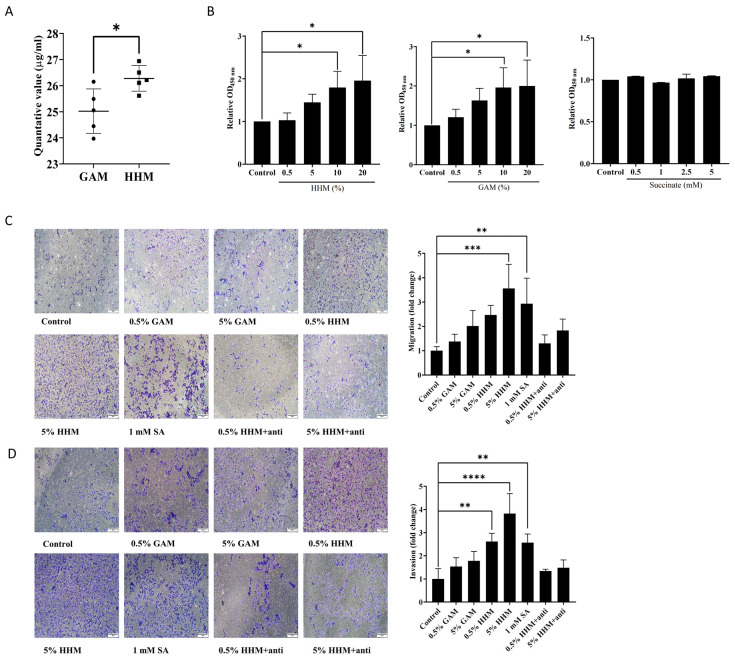
*H. hathewayi*-derived succinate could promote CRC metastasis. (**A**) Comparison of succinate levels between HHM and GAM. *H. hathewayi* was cultured at 37 °C for 48 h in GAM. HHM and fresh GAM medium (Control) were analyzed for targeted succinic acid quantification by LC-MS/MS. (**B**) Analysis of cell viability in HCT15 cells seeded in 96-well plates at a density of 10^4^ cells per well by CCK-8 assay. Cells were treated with cell culture media containing HHM, GAM (0.5%, 5%, 10%, and 20%), or SA (0.5 mM, 1 mM, 2.5 mM, and 5 mM). (**C**) HCT15 cells seeded in the upper chamber of the transwell plates were treated with HHM, GAM, HHM + anti, or SA for 48 h. HHM, GAM, and HHM + anti was used at 0.5% and 5% (*v*/*v*), while SA (positive control) was used at 1 mM. Untreated cells served as blank controls. The number of migrated cells is presented as fold change relative to the control group. Scale bars, 200 μm. (**D**) Cell invasion was analyzed using Matrigel-coated inserts. All other experimental conditions were as described in (**C**). Scale bars, 200 μm. HHM, the culture medium supernatant of *H. hathewayi*. GAM, bacterial culture medium. HHM + anti, HHM with anti-succinate antibodies. SA, succinate. Data were from independent experiments and shown as mean ± SD. Compared using Student’s *t*-test between two groups, and differences among the groups were calculated using One Way ANOVA, followed by Dunnett’s multiple comparison test. Differences among the groups were calculated using One Way ANOVA. * *p* < 0.05, ** *p* < 0.01, *** *p* < 0.001, and **** *p* < 0.0001.

Our results indicated that *H. hathewayi* can induce an inflammatory response in intestinal epithelial cells, which is associated with the development of CRC. It was reported that the succinate receptor SUCNR1 mediates the initiation of the inflammasome in intestinal epithelial cells. Therefore, we hypothesized that *H. hathewayi* could exert its effects through producing succinate to interact with SUCNR1. To investigate this hypothesis, we conducted a metabolomic analysis of HHM to detect succinate using thin-layer chromatography (TLC) and targeted organic acid assays. The TLC results confirmed the presence of succinate in the HHM (Supplementary Figure S3), while the LC-MS/MS analysis revealed that the succinate concentration in the HHM was significantly elevated compared to that in the bacterial culture medium (GAM) (Figure 4A).

Numerous studies have documented succinate’s role as a signaling molecule in intestinal injury and immune response [29,30,31]. Research data indicate elevated succinate levels in the feces of CRC patients compared to healthy individuals [23]. To investigate succinate’s impact on CRC development, we performed CCK-8 experiments, revealing that components in HHM and GAM facilitated CRC cell proliferation. To investigate the impact of succinate on CRC development, we performed CCK-8 assays. The results revealed that components present in HHM and GAM promoted CRC cell proliferation. However, this proliferative effect was not attributable to succinate; our findings were consistent with previous reports [32] (Figure 4B).

Furthermore, as SCUNR1 is a specific receptor for succinate, we added anti-succinate antibodies to the HHM. Our results indicated that both the HHM and succinate enhanced the migratory and invasive capabilities of CRC cells. In contrast, the groups treated with antibodies and those exposed to the bacterial culture medium did not exhibit similar effects (Figure 4C,D). Our results indicated that *H. hathewayi*-derived succinate can promote the migration and invasion abilities of CRC.

### 3.5. H. hathewayi-Derived Succinate Upregulates HIF-1α and SUCNR1 Expression and Promotes Metastasis by Inducing EMT in HCT15 Cells

**Figure 5:**
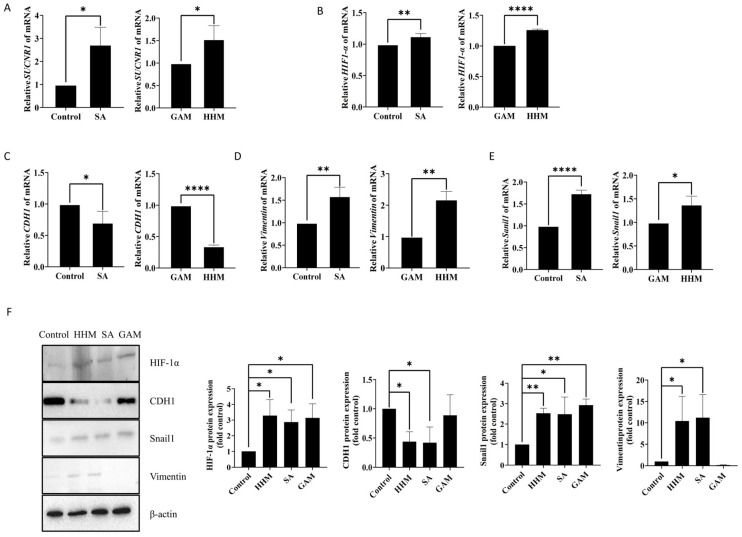
*H. hathewayi*-derived succinate upregulates *HIF-1α* and *SUCNR1* expression and promotes metastasis by inducing EMT in HCT15 cells. (**A**) Relative mRNA expression of *SUCNR1* in HCT15 cells treated with SA or HHM by qPCR. HCT15 cells were cultured in cell medium with 5% HHM, GAM, and SA (positive control) for 24 h. (**B**) Relative mRNA expression of *HIF-1α* in HCT15 cells treated with 1 mM SA or HHM by qPCR. Cells were cultured under conditions described in (**A**). Untreated cells served as the blank control. (**C**–**E**) Relative mRNA expression of *CDH1*, *Vimentin* and *Snail1* in HCT15 cells treated with SA or HHM by qPCR. Cells were cultured under conditions described in (**A**). Untreated cells served as the blank control. (**F**) Analysis of CDH1, HIF-1α, Snail1, and Vimentin protein expression in HCT15 cells by Western blot. HCT15 cells were cultured in cell medium with 5% HHM, GAM, and SA (positive control) for 24 h. HHM, the culture medium supernatant of *H. hathewayi*. GAM, bacterial culture medium. SA, 1 mM succinate. Data were from independent experiments and shown as mean ± SD. Compared using Student’s *t*-test between two groups, and differences among the groups were calculated using One Way ANOVA, followed by Dunnett’s multiple comparison test. Differences among the groups were calculated using One Way ANOVA. * *p* < 0.05, ** *p* < 0.01 and **** *p* < 0.0001.

We further confirmed the mechanism by which *H. hathewayi*-derived succinate promotes CRC metastasis. Real-time PCR revealed an increase in *SUCNR1* expression in HCT15 cells treated with HHM or succinate, suggesting that *H. hathewayi*-derived succinate exerted its effects through *SUCNR1* (Figure 5A). HIF-1α, a critical marker, has been reported in multiple studies to exhibit significantly higher expression levels in tumor tissues compared to normal tissues [33,34,35]. Additionally, succinate has been shown to facilitate lung cancer metastasis via EMT through the SUCNR1-mediated HIF-1α signaling pathway [32]. However, this pathway remains unverified in CRC.

Therefore, we further confirmed whether *H. hathewayi*-derived succinate could trigger EMT in the CRC cell line HCT15. Real-time PCR and Western bolt results showed that the expression of *HIF-1α* was upregulated in the HHM treatment group and the succinate treatment group (Figure 5B,F). Meanwhile, HHM and succinate can inhibit the expression of *E-cadherin* (*CDH1*) and increase the expression of *Vimentin* and EMT-transcription factor *Snail1* (Figure 5C–F). These results indicate that *H. hathewayi*-derived succinate upregulates *HIF-1α* and *SUCNR1* expression and promotes metastasis by inducing EMT in HCT15 cells.

## 4. Discussion

The human body hosts a vast array of symbiotic microorganisms that are crucial for regulating the immune system, nutritional metabolism, and host defense mechanisms. The advent of next-generation sequencing technology has challenged the traditional belief that tumors exist in sterile environments [36]. Research has revealed a diverse range of bacteria present in various tumor tissues and their adjacent areas [37]. These bacteria, as primary components of the microbiome, significantly influence different cancer types. For example, patients with oral cancer exhibit a notable increase in *Alloprevotella* abundance [38]. Additionally, *Alloprevotella*, *Porphyromonas gingivalis*, and *F. nucleatum* are enriched in Oral Squamous Cell Carcinoma (OSCC) tumor samples [39]. In gastric cancer, a high abundance of *Methylobacterium*, *Prevotella*, and *F. nucleatum* correlates with a reduced overall survival period [40,41]. These findings collectively suggest that specific bacterial types play critical roles in the onset, progression, and prognosis of tumors in various parts of the human body.

CRC ranks among the most lethal malignant tumors today, often diagnosed at mid to advanced stages, leading to high mortality rates and poor prognoses. Intestinal bacteria are essential for maintaining intestinal health and homeostasis; however, an imbalance in this flora can have detrimental effects. Recent studies have identified bacteria such as *F. nucleatum*, toxigenic *E. coli*, *P. anaerobius*, and *B. fragilis* as being closely linked to the pathogenicity of CRC [13,15,18,19,42,43,44,45,46,47]. In both CRC and other cancer types, different bacteria uniquely influence tumor development and progression. Despite the established connection between bacteria and cancer progression, and the detection of pathogenic bacteria within tumors, directly isolating and culturing these strains from tumor tissues to confirm their relationship with cancer remains challenging.

In this study, we attempted to tackle the previously mentioned issues, with a primary focus on the pathogenic strains associated with CRC. Utilizing culture omics, we isolated a strain of *H. hathewayi* from the tumor tissues of CRC patients. The reference strain *H. hathewayi* has been reported to be prevalent in both fecal samples and tumor tissues from CRC patients [25,26]. Additionally, studies show that inoculating mice with *H. hathewayi* stimulates the proliferation of colonic epithelial cells [27]. These results prompt us to further explore the role of tissue-resident *H. hathewayi*, directly isolated from primary tumor tissues, in affecting CRC progression.

In our study, we employed two pairs of specific primers for *H. hathewayi* and analyzed the abundance of *H. hathewayi* in the feces and tumor tissues of CRC patients through qPCR. We also created fluorescent probes and polyclonal antibodies against *H. hathewayi*. Using FISH, IF, and IHC, we observed the localization and quantification of *H. hathewayi* in various tissue sites. Our results demonstrated that *H. hathewayi* colonized tumor tissues in higher abundance than normal tissues. Furthermore, we collected fecal samples from CRC patients and healthy individuals to compare the abundance of *H. hathewayi* by qPCR. The findings revealed a significantly higher abundance of *H. hathewayi* in the feces of CRC patients compared to healthy individuals. This suggests that *H. hathewayi* could serve as a potential diagnostic marker for CRC. Given the challenges of early CRC detection, which often result in late-stage diagnosis and poor prognosis, monitoring *H. hathewayi* abundance could offer a practical approach for early detection and risk assessment during routine medical visits. Notably, several limitations of this study should be acknowledged. First, qPCR indicated an increasing trend in *H. hathewayi* abundance in non-tumor tissues adjacent to the cancer, but this increase was not statistically significant compared with the normal tissue (P48, *p* = 0.08; P52, *p* = 0.19). This may be due to a limited sample size affecting the statistical power or significant individual variability within the group obscuring potential trends. Second, the sample size used for ROC curve analysis to evaluate the diagnostic potential of fecal *H. hathewayi* was also relatively small. A limited sample cohort may lead to the overestimation of diagnostic accuracy and reduce the generalizability of the findings. Although we performed independent validation to confirm the robustness of the selected cutoff values, the validation cohort was modest in size. Therefore, larger, multicenter studies with more diverse populations are warranted to further validate the diagnostic performance of fecal *H. hathewayi* and to establish more reliable cutoff values before clinical application. Additionally, the short postoperative follow-up period and small patient cohort in this study precluded the analysis of the relationship between patient prognosis and *H. hathewayi*. Thus, further research is needed to explore the value of *H. hathewayi* as a prognostic marker for CRC. In future studies, we will continuously expand the number of clinical samples to address these limitations, with the goal of establishing *H. hathewayi* as a prognostic tumor marker for CRC, alongside *F. nucleatum*.

The SW620 cell line, established from a lymph node metastasis of colorectal carcinoma, serves as an important model for studying CRC and mimicking intestinal epithelial functions due to its retention of certain characteristic features of intestinal epithelial cells [48]. The significant presence of *H. hathewayi* in tumors prompts further investigation into its pathogenic role in CRC. Our initial findings reveal that *H. hathewayi* activates the NLRP3/ASC inflammasome in SW620 cells, leading to the activation of Caspase-1 and the subsequent release of IL-1β and IL-18. This suggests that in a model simulating the intestinal epithelial environment, *H. hathewayi* can damage these cells and provoke the release of inflammatory factors, leading to a localized inflammatory response. Additionally, using TLC and LC-MS/MS targeted organic acid detection, we confirmed that *H. hathewayi* secretes succinate. It has been reported that the succinate receptor SUCNR1 mediates the priming step of the inflammasome in intestinal epithelial cells like HT-29 [49]. In the tumor microenvironment, bacteria predominantly modulate the host’s inflammatory response through continuous succinate secretion. Based on our findings, we hypothesize that *H. hathewayi* similarly secretes succinate, which, through SUCNR1 mediation, activates NLRP3 in SW620 cells, resulting in the release of IL-1β and IL-18.

Building on these results, we concentrated on succinate, a metabolite of *H. hathewayi*, as our research focus. CCK-8 assays indicated that succinate did not enhance CRC cell proliferation, aligning with previous findings [32]. However, we observed that succinate significantly promoted the migration of HCT15 cells. Additionally, treatment with HHM and succinate led to an upregulation of *HIF-1α* mRNA expression in HCT15 cells. Concurrently, the expression levels of EMT-related genes, including *CDH1*, *Vimentin*, and *Snail1*, were altered. Our results demonstrate that *H. hathewayi*-derived succinate can induce EMT in the CRC cell line HCT15 and enhance cell metastasis by upregulating *HIF-1α* expression via *SUCNR1*.

A recent study highlighted a significant link between succinate and intestinal microbiota in ulcerative colitis (UC). Succinate consumption markedly boosts probiotic levels in the intestinal tract of mice and increases the *Firmicutes*-to-*Bacteroidetes* ratio compared to the inflammatory group [50]. This suggests that succinate serves not only as a crucial metabolic product in the intestinal microenvironment but also as a vital signaling molecule that directly influences microbiota structure and the host–microbiota balance. A similar phenomenon has been reported in CRC: succinic acid levels were elevated in the feces of CRC patients compared with healthy subjects [23]. Importantly, we found that the *H. hathewayi* strain is enriched in the fecal samples of CRC patients compared to healthy controls. Given this strain’s demonstrated ability to secrete succinate and its enrichment in CRC patient feces, it may be one of the contributors to the elevated fecal succinate levels in CRC patients.

Succinate, a crucial signaling molecule, significantly influences the regulation of the tumor immune microenvironment (TIME). Notably, cancer-derived succinate dose-dependently upregulates ARG1 protein and TAM marker gene expression (Arg1, Fizz1, MgI1, and MgI2) in mouse peritoneal macrophages and promotes the differentiation toward a VCAM1^+^CD11c^+^CD11b^low^ tumor-associated macrophage (TAM) phenotype [32]. In vivo, succinate treatment likewise increases the infiltration ratio of TAM subpopulations in primary tumor tissues [32]. Additionally, *F. nucleatum*-derived succinate suppresses the cGAS-IFN-β signaling pathway, which reduces the tumor expression of CCL5 and CXCL10. As a result, CD8^+^ T-cell infiltration into the tumor microenvironment is limited, weakening the efficacy of anti-PD-1 therapy [23]. Collectively, these studies show that succinate in the TIME both promotes an immunosuppressive milieu by altering macrophage phenotypes and impairs adaptive immunity by disrupting T-cell recruitment. Notably, our isolated strain, *H. hathewayi*, is enriched in CRC tumor tissue and secretes succinate, indicating that it could be a direct intratumoral source of succinate. Thus, *H. hathewayi* may act on the tumor immune microenvironment to promote immune suppression via the mechanisms described above. Targeting the succinate signaling axis could therefore be a promising strategy to reverse tumor immunosuppression and enhance responses to immunotherapy.

In summary, we isolated the strain *H. hathewayi* from the tumor tissues of CRC patients, identifying its significant colonization and abundance in these tissues. Functional studies revealed that *H. hathewayi*-derived succinate upregulates the expression of *HIF-1α* and *SUCNR1* in HCT15 cells and promotes cell metastasis by inducing EMT. These findings provide new insights into the pathogenic mechanisms of *H. hathewayi* in CRC progression. Additionally, its presence in both the feces and tumor tissues of CRC patients suggests that *H. hathewayi* could serve as a potential tumor marker for CRC. This discovery may offer new target potentials and perspectives for clinical diagnosis.

## Figures and Tables

**Table 1:** Sequences of primers used in qPCR.

Gene	Forward Primer (5′ to 3′)	Reverse Primer (5′ to 3′)
*NLRP3*	ACAAACTCATGGTGGCTTCC	GGCCAGAAGAAAAGCAAGTG
*IL-1β*	ATGATGGCTTATTACAGTGGCAA	GTCGGAGATTCGTAGCTGGA
*ASC*	TGACGGATGAGCAGTACCAG	AGGATGATTTGGTGGGATTG
*Caspase-1*	CGCACACGTCTTGCTCTCAT	TACGCTGTACCCCAGATTTTGTAG
*IL-18*	GATAGCCAGCCTAGAGGTATGG	CCTTGATGTTATCAGGAGGATTCA
*HIF-1α*	TGCTCATCAGTTGCCACTTC	TCCTCACACGCAAATAGCTG
*SUCNR1*	GGGAGTTGGAAGCAGTATCAG	GCATGTCCCTGAAGTGATCTC
*CDH1*	ATTTTTCCCTCGACACCCGAT	TCCCAGGCGTAGACCAAGA
*Vimentin*	AGTCCACTGAGTACCGGAGAC	CATTTCACGCATCTGGCGTTC
*Snail1*	TCCAGAGTTTACCTTCCAGCA	CTTTCCCACTGTCCTCATCTG
*β-actin*	CACCATTGGCAATGAGCGGTTC	AGGTCTTTGCGGATGTCCACGT

## Data Availability

The original contributions presented in this study are included in the article/Supplementary Material. Further inquiries can be directed to the corresponding authors.

## Supplementary Materials

The following supporting information can be downloaded at: https://www.mdpi.com/article/10.3390/microorganisms14030707/s1; Figure S1: *H. hathewayi* abundance in early-stage and late-stage CRC tumor tissues; Figure S2: Validation of polyclonal antibody specificity; Figure S3: Verification of succinate production from *H. hathewayi* via TLC; Table S1: Diagnostic performance of fecal *H. hathewayi* detection using primer P48 in discovery and validation cohorts; Table S2: Diagnostic performance of fecal *H. hathewayi* detection using primer P52 in discovery and validation cohorts; Table S3: Clinical information of patients and healthy controls (fecal samples); Table S4: Clinical information of patients and healthy controls (tissue samples).
