# Supplementary material for: Hungatella hathewayi: A Tumor-Derived Bacterium Enriched in Colorectal Cancer Tissues and a Potential Diagnostic Biomarker
Source: Microorganisms. 2026 Mar 21;14(3):707. doi: 10.3390/microorganisms14030707 (PMC13028988; doi:10.3390/microorganisms14030707)
Supplement: Supplementary file 1 [file microorganisms-14-00707-s001.zip › Supplementary Figure S1-S3.pdf]

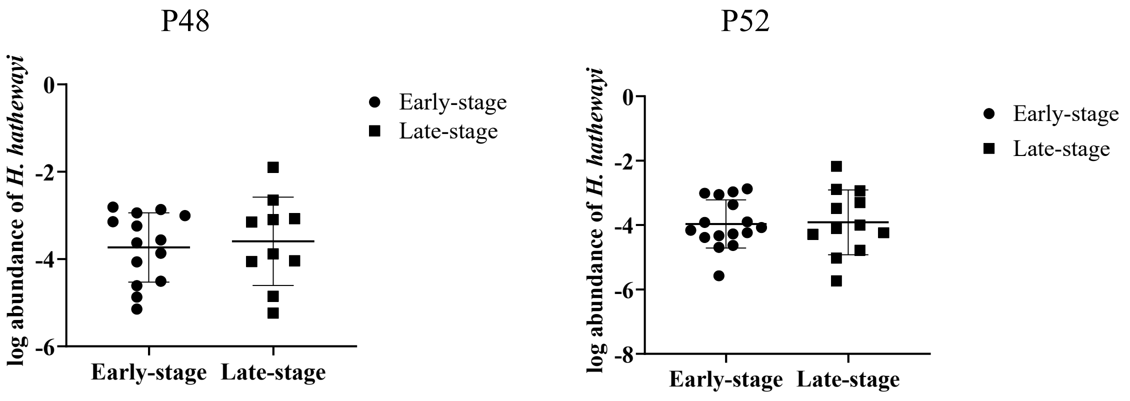

Figure S1 *H. hathewayi* abundance in early-stage and late-stage CRC tumor tissues. qPCR analysis using primers P48 and P52 revealed no significant difference in *H. hathewayi* abundance between early-stage (I–II) and late-stage (III–IV) CRC tumor tissues. Data are presented as mean ± SD.

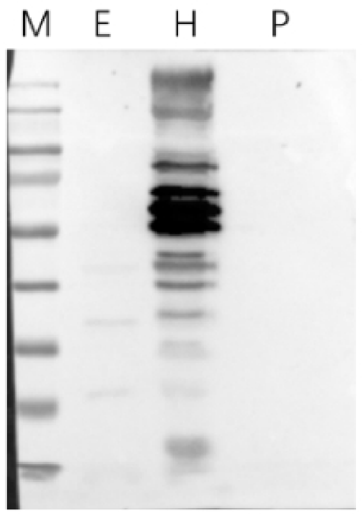

Figure S2 Validation of polyclonal antibody specificity. Western blot analysis of the specificity of polyclonal antibody. M: protein marker, E: *E.coli*, H: *H. hathewayi*, P: *P. anaerobius*

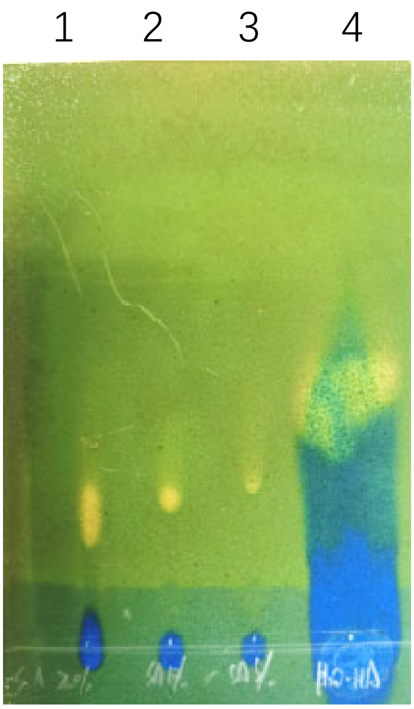

Figure S3 Verification of succinate production from *H. hathewayi* via TLC. Lane 1: 1.69 M succinate. Lane 2, 0.87 M succinate. Lane 3, 0.43 M succinate. Lane 4, Water-soluble lyophilized powder.
