# Supplementary material for: Hungatella hathewayi: A Tumor-Derived Bacterium Enriched in Colorectal Cancer Tissues and a Potential Diagnostic Biomarker
Source: Microorganisms. 2026 Mar 21;14(3):707. doi: 10.3390/microorganisms14030707 (PMC13028988; doi:10.3390/microorganisms14030707)
Supplement: Supplementary file 1 [file microorganisms-14-00707-s001.zip › Supplementary Table S1 and S2.pdf]

Supplementary Table S1. Diagnostic performance of fecal *H. hathewayi* detection using primer P48 for CRC identification in discovery and validation cohorts

	**Discovery cohort**	**Validation cohort**
Cutoff value	3.19×10^{-5}	3.19×10^{-5}
Area under the curve	0.8871*	-
Sensitivity (%)	85	85 (17/20)
Specificity (%)	91.43	91.43 (32/35)
Sample size (CRC/Healthy)	20/35	20/35

* 95% confidence interval (CI): 0.7773–0.9970

Supplementary Table S2. Diagnostic performance of fecal *H. hathewayi* detection using primer P52 for CRC identification in discovery and validation cohorts

	**Discovery cohort**	**Validation cohort**
Cutoff value	3.55×10^{-5}	3.55×10^{-5}
Area under the curve	0.8872*	-
Sensitivity (%)	78.95	78.95 (17/19)
Specificity (%)	88.57	88.57 (31/35)
Sample size (CRC/Healthy)	19/35	19/35

* 95% confidence interval (CI): 0.7770–0.9974
